# Toll-Like Receptors Expressed by Synovial Fibroblasts Perpetuate Th1 and Th17 Cell Responses in Rheumatoid Arthritis

**DOI:** 10.1371/journal.pone.0100266

**Published:** 2014-06-17

**Authors:** Fanlei Hu, Yingni Li, Li Zheng, Lianjie Shi, Hongjiang Liu, Xuewu Zhang, Huaqun Zhu, Sumei Tang, Lei Zhu, Liling Xu, Yuqin Yang, Zhanguo Li

**Affiliations:** Department of Rheumatology and Immunology, Peking University People’s Hospital, Beijing, China; Universidade Federal do Rio de Janeiro, Brazil

## Abstract

Rheumatoid arthritis (RA) is a chronic inflammatory disease characterized by synovial fibroblast hyperplasia and bone and cartilage erosion. Synovial fibroblast- and T cell-mediated inflammation plays crucial roles in the pathogenesis of RA. However how this inflammation is initiated, propagated, and maintained remains controversial. Here, we systemically examined the contribution of toll-like receptors (TLRs) to the inflammatory mediator production as well as Th1 and Th17 cell hyperactivity in RA. Our results show that rheumatoid arthritis synovial fibroblasts (RASF) express a series of TLRs, including TLR2, TLR3, TLR4, and TLR9, with the predominant expression of TLR3. Moreover, the expression levels of these TLRs were higher than those in osteoarthritis synovial fibroblasts (OASF). Ligation of TLR3, as well as TLR2 and TLR4, resulted in vigorous production of inflammatory cytokines, matrix metalloproteinases (MMPs), and vascular endothelial growth factor (VEGF) in RASF, with activation of the NF-κB, MAPK, and IRF3 pathways. More important, activation of these TLRs expressed by RASF exacerbated inflammatory Th1 and Th17 cell expansion both in cell-cell contact-dependent and inflammatory cytokine-dependent manners, which induced more IFN-γ and IL-17 accumulation. Targeting TLRs may modulate the inflammation in RA and provide new therapeutic strategies for overcoming this persistent disease.

## Introduction

Rheumatoid arthritis (RA) is a chronic autoimmune inflammatory disease that causes deformity of the joints and physical disability. It affects approximately 1% of the population, and up to 30% of RA patients become permanently work disabled within 3 years of diagnosis if they do not receive medical treatment [1], [2].

One of the hallmarks of RA is a dysregulated activation of the inflammatory responses, particularly the Th1 and Th17 responses. Elevated levels of proinflammatory cytokines were demonstrated in RA patient sera and synovial fluids, such as IL-6, IL-8, TNF-α, IL-17, and IL-33 [3], [4], [5]. TNF-α monoclonal antibodies have long been proved to be able to dramatically decrease signs and symptoms of RA. Therapeutic strategies targeting IL-6 also benefited many RA patients [6], [7]. However, how the inflammation in RA is initiated and perpetuated remains controversial.

Recent evidence suggested that rheumatoid arthritis synovial fibroblasts (RASF) are one of the most important cells contributing to RA pathogenesis. They are potent participants in all aspects of the initiation, progression, and perpetuation of the disease. The hyperproliferated RASF in the synovium are aggressive to attack cartilage by secreting matrix metalloproteinases (MMPs), which would eventually lead to bone erosion and destruction [8]. More important, RASF could act as effectors of innate immunity to secrete a broad array of inflammatory mediators: cytokines (such as IL-6, IL-8, and TNF-α), chemokines (such as MCP-1, MIP-1α, and IP-10), and several other proinflammatory molecules like prostaglandins and leukotrienes. And RASF in the intimal lining were reported to be the primary source of IL-6 in RA [3], [9]. In addition, RASF could interact with T cells through cell-cell contact and/or inflammatory cytokines, which induces inflammatory T cell expansion, thus exacerbating the inflammation in RA. It is of significance to understand how RASF and T cells become actived to perpetuate the inflammation in RA patients.

Exposure of cytokines and ligation of integrins by matrix molecules account for RASF and T cell activation partly. Additionally, our group as well as other groups revealed that hypoxia and hypoxia-inducible factor-1α (HIF-1α) potentiated the production of inflammatory cytokines, MMPs, and vascular endothelial growth factor (VEGF) by RASF. Moreover, HIF-1α overexpression promoted RASF-mediated inflammatory T cell expansion [5], [10], [11], [12]. Recently, the role of toll-like receptors (TLRs) in RA has also been recognized gradually [13], [14], [15], [16], [17], [18]. TLRs are a class of proteins that play a fundamental role in the innate immune system. They recognize “pathogen-associated molecular patterns (PAMP)” which are structurally conserved molecules derived from microbes and are distinguishable from host molecules, and activate the innate immune responses. Till now, more than 13 members of the TLR family have been identified in mammals [19], [20]. To define the expression profile of TLRs and their pathogenic roles will improve our understanding of RA.

In this study, we systemically detected the expression of TLRs in RA, and revealed their roles in perpetuating the persistent inflammation.

## Materials and Methods

### Patients, Tissue Specimens, and Ethics Statement

Synovial tissue specimens used for culturing RASF (n = 5) and osteoarthritis synovial fibroblasts (OASF, n = 5) were obtained from patients during total knee replacement surgery or arthroscopy. All patients fulfilled the American College of Rheumatology 2009 criteria for RA and 1995 criteria for OA, and provided their written consents to participate in this study. The study protocols, consent forms, and consent procedure were approved by the Institutional Medical Ethics Review Board of Peking University People’s Hospital.

### Cell Culture and Stimulation

RASF and OASF were cultured as previously described [5], and were used at passages 4–6. For TLR stimulation, 5×10^4^ RASF per well in 6-well plates were incubated with different TLR ligands for 4 h and 24 h, respectively, as follows: TLR2 ligand peptidoglycan (PGN, 10 µg/ml), TLR3 ligand polyinosinic:polycytidylic acid (ploy(I:C), 25 µg/ml), TLR4 ligand lipopolysaccharide (LPS, 100 ng/mL) from Sigma; TLR9 ligand Type B CpG (10 µg/ml) from Invitrogen (Carlsbad, CA, USA). The cells were harvested for RT-PCR and realtime PCR while the supernatants were collected for ELISA.

### RASF/T cell Co-culture Assay

5×10^4 ^RASF per well in 6-well plates were stimulated with or without 25 µg/ml ploy(I:C) or 100 ng/ml LPS for 24 h. Then the cells were washed with serum-free RPMI 1640 and co-cultured with 2.5×10^5^ allogeneic flow cytometry-sorted CD4^+^ RA patient T cells (>99% purity) at the ratio of 1∶5 in the presence of anti-CD3 and anti-CD28 (3 µg/ml each), with or without a 0.4-µm-pore-size membrane. Sometimes, the co-culture was performed under Th17 polarization conditions: 3 µg/ml anti-CD3, 3 µg/ml anti-CD28, 1 µg/ml anti-IFN-γ, 2.5 µg/ml anti-IL-4, 2.25 ng/ml TGF-β1, 30 ng/ml IL-6, 20 ng/ml IL-1β, and 30 ng/ml IL-23. 5 days later, the T cells were harvested for flow cytometry while the cell culture supernatants were collected for ELISA.

### RT-PCR and Realtime PCR

Total RNA was extracted from cells using TRIzol reagent (Invitrogen) and was treated with TURBO DNase (Ambion, Austin, TX, USA) to eliminate genomic DNA contamination. Reverse transcription was performed with the RevertAid First Strand cDNA synthesis kit (Fermentas, Glen Burnie, MD, USA) according to the manufacturer’s instructions. The resulting cDNA was subjected to PCR and realtime PCR analyses.

PCR was performed to analyze the expression of TLRs and inflammatory mediators in RASF. The primers used for amplification of TLR2 and TLR4 were as follows: TLR2 sense primer: 5′-ACCAAGTGAAGGTACCTGTGGGGC-3′, antisense primer: 5′-GCACCAGAGCCTGGAGGTTCAC-3′; TLR4 sense primer: 5′-CCCCGACAACCTCCCCTTCTCA-3′, antisense primer: 5′-TCCAGAAAAGGCT CCCAGGGCT-3′. The primers for TLR3, TLR9, IL-6, IL-8, TNF-α, and MMP-1 were the same as those used in our previous studies [12], [21]. The PCR products were separated by gel electrophoresis on 1% agarose.

Two-step realtime PCR was also performed using SYBR Green Master Mix (Applied Biosystems, Foster City, CA, USA) according to the manufacturer’s instructions. The primers for TLR2, TLR4, and TLR9 were the same as those used for PCR. The primers for TLR3, thrombospondin 1 (TSP-1), stromal cell-derived factor 1 (SDF-1), and IL-15 were as follows: TLR3 sense primer: AGGAAAGGCTAGCAGT CATCC, antisense primer: AGCAACTTCATGGCTAACAGTG; TSP-1 sense primer: 5′-CCTATGCTGGTGGTAGACTA-3′, antisense primer: 5′-ACGTTCTAGGAGTCC ACACT-3′; SDF-1 sense primer: 5′-ACACTCCAAACTGTGCCCTTCA-3′, antisense primer: 5′-CCACGTCTTTGCCCTTTCATC-3′; IL-15 sense primer: 5′-TTCACTTG AGTCCGGAGATGC-3′, antisense primer: 5′-GCATCCAGATTCTGTTACATTCC C-3′. The other primers were as previously reported [12]. Gene expression was quantified relative to the expression of the housekeeping gene GAPDH, and normalized to control by standard 2^−ΔΔCT^ calculation.

### Western Blot Analysis

RASF were stimulated with 25 µg/ml TLR3 ligand ploy(I:C) for 0, 5, 15, and 30 min, respectively. Then the cells were collected for western blot analysis as described previously [5]. The antibodies used were as follows: anti-phospho-IκBα mAb, anti-phospho-ERK mAb, anti-phospho-p38 mAb, anti-phospho-IRF3 mAb, anti-IκBα mAb, anti-ERK mAb, anti-p38 mAb, and anti-IRF3 mAb from Cell Signalling Technology (Danvers, MA, USA); anti-actin mAb from Tianjin Sungene Biotech Co., Ltd (Tianjin, China).

### Flow Cytometry Analysis

RA patient T cells were co-cultured with RASF for 5 days as described above. At the end of co-culture, the cells were stimulated with PMA (50 ng/ml) plus ionomycin (1000 ng/ml) for 5 h in the presence of Brefeldin A (10 µg/ml). Then, the cells were stained with PerCP-CY5.5–conjugated anti-CD4, fixed and permeabilized, followed by intracellular staining using FITC-conjugated anti-IFN-γ, and Alexa Fluor 647-conjugated anti-IL-17 (eBioscience, San Diego, CA, USA). Percentage of positive cells was analyzed on a FACS Arial II flow cytometer (Becton Dickinson, San Diego, CA, USA).

### ELISA Assay

Commercially available ELISA kits used for measuring inflammatory cytokines and MMPs were as follows: IL-6, IL-8, and IFN-γ ELISA kits from Neobioscience Technology Co, Ltd (Beijing, China); IL-17 ELISA kit from QuantoBio Biotechnology Co, Ltd (Beijing, China); TNF-α ELISA kit from eBioscience; and MMP-3 ELISA kit from R & D Systems (Minneapolis, Minnesota, USA).

### Statistical Analysis

SPSS 17.0 (SPSS, Chicago, Illinois, USA) was used for statistical analysis. Differences between various groups were evaluated by Wilcoxon signed-rank test, and were considered statistically significant when *P* was <0.05.

## Results

### Excessive Expression of Functional TLRs in RASF

**Figure 1:**
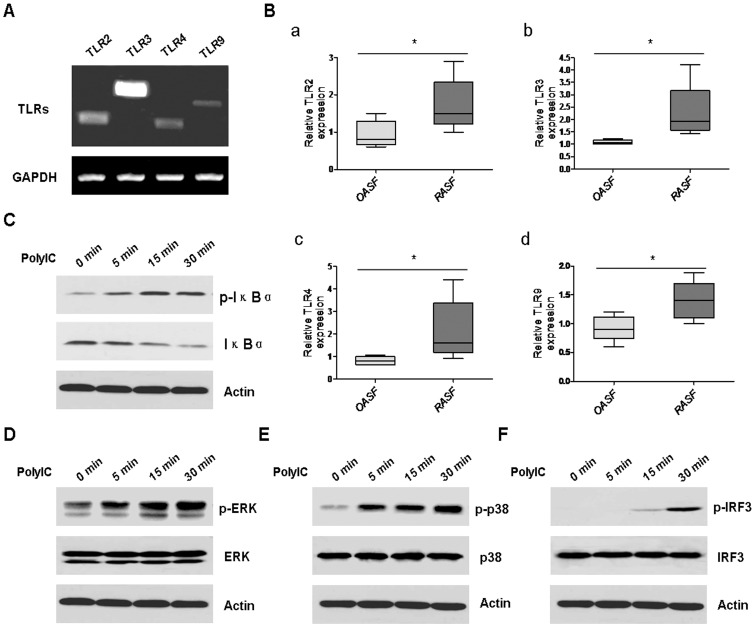
Functional TLRs were excessively expressed in RASF. A, The expression of TLRs, including TLR2, TLR3, TLR4, and TLR9 in RASF was detected by RT-PCR analysis. GAPDH was used as the loading control. B, The expression of TLR2, TLR3, TLR4, and TLR9 in RASF (n = 5) and OASF (n = 5) was determined by realtime PCR (Wilcoxon signed-rank test, *P<0.05). RASF were stimulated with 25 µg/ml ploy(I:C) for 5 min, 15 min, and 30 min, respectively. Then the cells were harvested and lysised for western blot analysis of p-IκBα (C), p-ERK (D), p-p38 (E), and p-IRF3 (F). β-actin was used as the loading control. The results were representative of at least three experiments.

We first detected the expression of TLRs in RASF by RT-PCR. As shown in Figure 1A, different TLRs, including TLR2, TLR3, TLR4, and TLR9 were present in RASF, with the highest expression levels of TLR3 and the lowest expression levels of TLR9. Realtime PCR further demonstrated that the expression levels of these TLRs were higher in RASF than in OASF (Figure 1B, a–d). To confirm these TLRs were functionally active, we stimulated RASF with TLR3 ligand ploy(I:C), and then examined the downstream signalling pathway responses, particularly the activation of the three most important pathways, NF-κB pathway, MAPK pathway, and IRF3 pathway. Our results demonstrated that after ploy(I:C) stimulation, the NF-κB pathway (Figure 1C), the MAPK pathway, including the ERK pathway (Figure 1D) and p38 pathway (Figure 1E), as well as the IRF3 pathway (Figure 1F) became activated, which could be seen from 5 minutes until 30 minutes after stimulation.

### TLR Ligation Potentiated Inflammatory Cytokine Production in RASF

**Figure 2:**
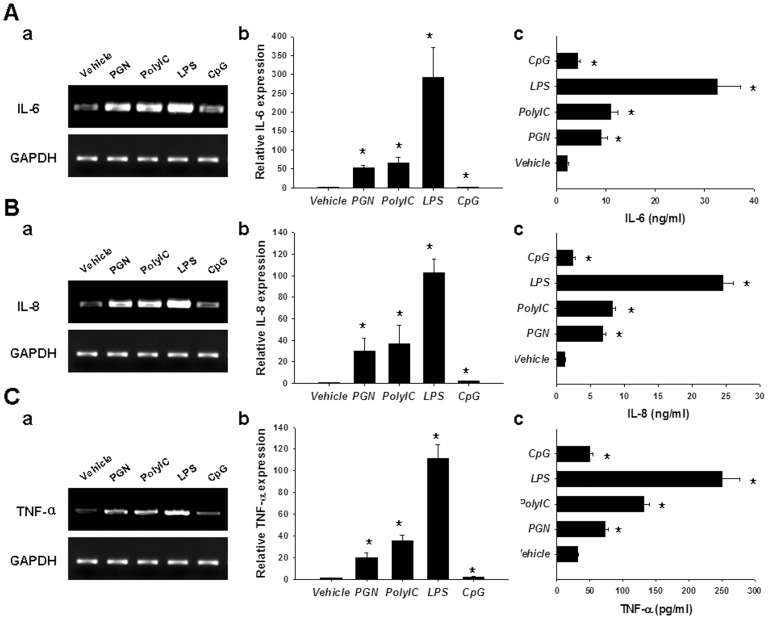
TLR agonists promoted RASF to produce inflammatory cytokines. RASF were stimulated with TLR2 agonist PGN (10 µg/ml), TLR3 agonist ploy(I:C) (25 µg/ml), TLR4 agonist LPS (100 ng/mL), or TLR9 agonist CpG (10 µg/ml). 4 h later, the cells were harvested for RT-PCR (A–C, a) and realtime PCR (A–C, b) analyses of IL-6, IL-8, and TNF-α; 24 h later, the cell culture supernatants were collected for ELISA analyses of IL-6, IL-8, and TNF-α (A–C, c). The results were presented as mean+SEM of five independent experiments (Wilcoxon signed-rank test, *P<0.05 *vs.* vehicle control).

To reveal the contribution of TLRs to the inflammation in RA, we stimulated RASF with different TLR ligands, and then detected the corresponding inflammatory cytokine production. Ligation of TLR2 with PGN, TLR3 with ploy(I:C), and TLR4 with LPS resulted in robust inflammatory cytokine production, including IL-6 (Figure 2A), IL-8 (Figure 2B), and TNF-α (Figure 2C), as demonstrated by RT-PCR (a), realtime PCR (b), and ELISA (c) analyses. Nonetheless, ligation of TLR9 with CpG merely induced weak production of these inflammatory cytokines (Figure 2A–C), which may be attributed to the relatively low expression levels of TLR9 in RASF.

### TLR Ligands Stimulated RASF to Produce MMPs and VEGF

**Figure 3:**
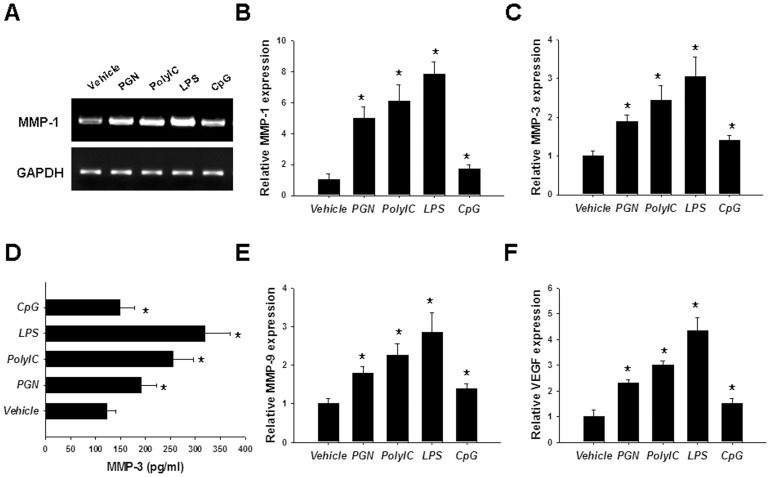
vLigation of TLRs stimulated MMPs and VEGF production in RASF. RASF were stimulated with 10 µg/ml PGN, 25 µg/ml ploy(I:C), 100 ng/mL LPS, or 10 µg/ml CpG for 4 h. Then the cells were harvested for RT-PCR analysis of MMP-1 (A); realtime PCR analyses of MMP-1 (B), MMP-3 (C), MMP-9 (E), and VEGF (F). And also, after stimulation for 24 h, the cell culture supernatants were collected for MMP-3 ELISA (D). The results were presented as mean+SEM of five independent experiments (Wilcoxon signed-rank test, *P<0.05 *vs.* vehicle control).

Cartilage destruction, bone erosion, and angiogenesis are also important phenotypes of RASF which are characterized by producing large amounts of MMPs and VEGF. So, we further determined the effects of TLR ligation on RASF-mediated MMP and VEGF production. RT-PCR showed that TLR ligands, particularly PGN, ploy(I:C), and LPS could stimulate RASF to produce MMP-1 (Figure 3A), which was further confirmed by realtime PCR analysis (Figure 3B). Similar results were also seen for MMP-3 (Figure 3C, D), MMP-9 (Figure 3E), and VEGF (Figure 3F) production as revealed by realtime PCR and ELISA analyses. All these results suggest that RASF could express functionally active TLRs.

### TLR Activation Exacerbated RASF-mediated Inflammatory Th1 and Th17 Cell Expansion

**Figure 4:**
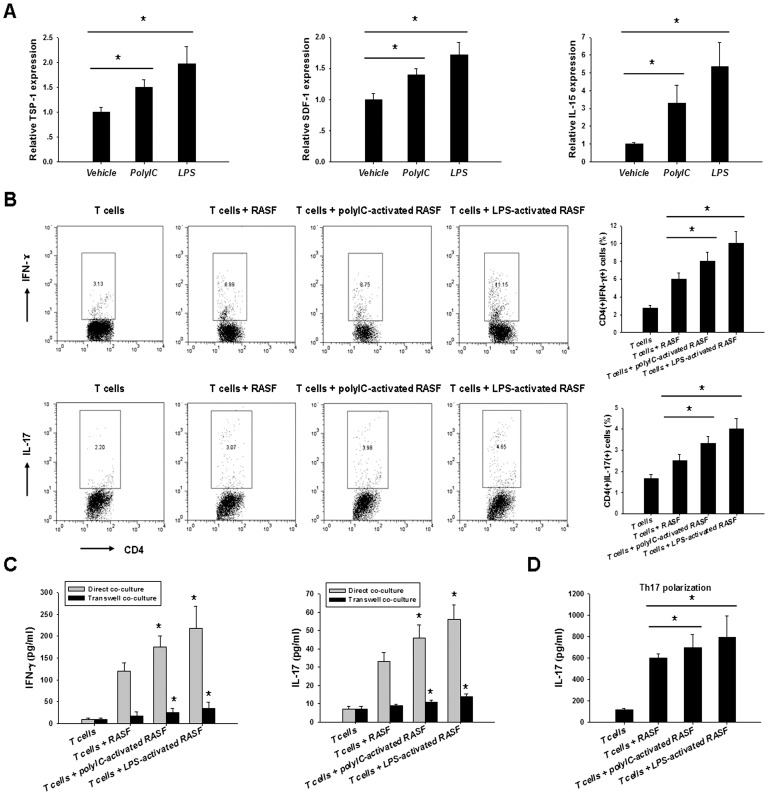
TLR activation enhanced RASF-mediated inflammatory Th1 and Th17 responses. A, RASF were stimulated with 25 µg/ml ploy(I:C) or 100 ng/ml LPS for 24 h, then the expression levels of TSP-1, SDF-1, and IL-15 were determined by realtime PCR analyses (Wilcoxon signed-rank test, *P<0.05). B, After activation with 25 µg/ml ploy(I:C) or 100 ng/ml LPS for 24 h, RASF were co-cultured with allogeneic CD4^+^ RA patient T cells in the direct contact or the transwell systems for 5 days. Then the T cells were harvested for flow cytometry analysis of the percentage of Th1 and Th17 cells (Wilcoxon signed-rank test, *P<0.05). Accordingly, the cell culture supernatants were collected for ELISA analyses of IFN-γ and IL-17 (C) levels (Wilcoxon signed-rank test, *P<0.05 *vs.* unstimulated control). D, RASF/T cell co-culture was performed as described above under Th17 polarization conditions. 5 days later, the cell culture supernatants were collected for IL-17 ELISA (Wilcoxon signed-rank test, *P<0.05). The data were shown as mean+SEM of four separate assays.

RASF could inhibit the apoptosis of T cells through cell–cell interaction and/or the elaboration of soluble factors, which elicits spontaneous proliferation of inflammatory T cells. The increased inflammatory T cells then in turn induce more robust RASF activation, thus forming a RASF/T-cell regulatory circuit that perpetuates the inflammation in RA [22]. So we next determined the impact of TLR activation on RASF-mediated inflammatory T cell expansion. After stimulation with ploy(I:C) or LPS for 24 h, the molecules mediating RASF-T interactions, including TSP-1, SDF-1, and IL-15, were upregulated in RASF (Figure 4A). When co-cultured with RA patient T cells for 5 days, these RASF induced more inflammatory Th1 and Th17 cells than the unstimulated ones, as shown by flow cytometry analysis (Figure 4B). Accordingly, higher levels of IFN-γ and IL-17 were seen in the cell culture supernatants (Figure 4C). More interestingly, this effect could also be seen when the cells were co-cultured under Th17 polarization conditions (Figure 4D). Cell-cell contact and inflammatory cytokines were both involved in this inflammation exacerbation. As shown in Figure 4C, comparable proportional increase of IFN-γ and IL-17 in ploy(I:C)- or LPS-stimulated cultures was seen in the direct contact and transwell systems. The absolute concentration differences may be attributed to the allogenic responses of RASF and T cells. However, no or little production of IL-10 and IL-4 which are potent anti-inflammatory cytokines in RA was detected after RASF/T co-culture (data not shown). Taken together, all these suggest that TLR activation could enhance RASF-mediated expansion of inflammatory T cells.

## Discussion

In this study, we revealed the distinct expression pattern of TLRs in RA. These receptors, particularly TLR2, TLR3, and TLR4 played crucial roles in the production of inflammatory cytokines, MMPs, and VEGF by RASF. More important, ligation of these TLRs expressed by RASF promoted inflammatory Th1 and Th17 responses. Thus TLRs contribute not only to the initiation but also to the perpetuation of the inflammation in RA which would be served as new therapeutic targets for the disease.

One of the most significant characteristics of RA is the intensive inflammation that is out of control. Managing to control the inflammation could prevent the disease progression, which would be the optimal strategy for RA therapy. One direct strategy is targeting the pathogenic inflammatory cytokines. Indeed, inhibitors of TNF-α have greatly advanced the management of RA, dramatically decreasing signs and symptoms of the diseases. At present, at least three agents are available which differ in pharmacokinetics and ability to bind lymphotoxin, crosslink membrane-bound TNF and induce apoptosis [6], [23]. Recently, IL-6 has also been proved to be a therapeutic target for RA. IL-6R monoclonal antibody tocilizumab showed promising perspective in treating RA patients [7]. Biological reagents targeting other inflammatory cytokines would appear in the following studies. However, RA is a complicated disease involving many inflammatory cytokines. So, targeting the upstream sources of the inflammation would demonstrate better efficacy for RA therapy.

Nonetheless, how the inflammation in RA is initiated, propagated, and maintained remains controversial. Infection has long been speculated to be an underlying factor contributing to RA pathogenesis. Indeed, peptidoglycan, bacterial and viral DNA as well as viral RNA were proved to be present in RA patient inflamed joints [24], [25]. These PAMPs would activate the corresponding TLRs, inducing robust inflammatory mediator production. In addition, endogenous heat shock protein, fibrinogen, hyaluronan, and double stranded RNA from apoptotic cells also exist in RA joints [13], [25]. These “danger-associated molecular patterns (DAMP)” could also provoke TLRs to induce inflammation. In this study, we proved that peptidoglycan (PGN), ploy(I:C) (a synthetic mimic of double stranded RNA from virus or apoptotic cells), LPS, and CpG (a synthetic mimic of DNA from bacteria or apoptotic cells) could activate RASF to induce vigorous inflammatory mediator production. This suggests that TLRs serve as an important contributor to the initiation and propagation of the inflammatory responses in RASF, which would eventually exacerbate the inflammation in RA.

In addition, in the current study, we showed for the first time that TLR activation exacerbated RASF-mediated inflammatory Th1 and Th17 responses. Th1 cells are considered to be the conventional pathogenic cells in RA, because RA is thought to be a Th1-biased disease with Th1/Th2 imbalance. Recently, attention has increasingly focused on the role of Th17 cells, a subset that produces IL-17A, 17F, 21, and 22 and TNF-α [26]. IL-17A, which synergizes with TNF-α to promote activation of fibroblasts and chondrocytes, is currently being targeted in clinical trials [27]. In RA, synovial fibroblasts and T cells form a co-stimulatory circuit to perpetuate the inflammation. RASF could inhibit the apoptotic process of T cells and elicit their spontaneous proliferation; the increased inflammatory T cells then in turn induce more robust RASF activation. Cell-cell contact and soluble factors, such as inflammatory cytokines, contribute to this interaction. As demonstrated in our study, after TLR ligation, RASF could secrete more inflammatory mediators and express higher levels of surface TSP-1, SDF-1, and IL-15. These factors together with other unknown elements may eventually lead to the amplification of Th1 and Th17 cells. Thus TLRs also contribute to the maintenance and perpetuation of the inflammation in RA.

Given the integral roles of TLRs in the initiation, propagation, and perpetuation of the inflammation in RASF as well as T cells, targeting TLRs would be a preferred therapeutic strategy. In fact, several groups have been testing the feasibility of this hypothesis by both animal and human studies. In a serum-transfer model, disease duration was shortened in TLR4 null mice [28]. In the pristane-induced arthritis rat model, TLR3 expression was significantly upregulated during early disease stages. TLR3 agonist stimulation augmented the disease severity, and small interfering RNA (siRNA) targeting TLR3 in vivo reduced disease severity [29]. The recombinant analogue of chaperonin 10, XToll, targeting TLR4 developed by Cbio Ltd, is now being tested in a phase II clinical trial for RA treatment given by subcutaneous injection. Antibodies targeting TLR2, OPN-305 and OPN-301, have been proved to be able to abrogate spontaneous cytokine release by RASF [14]. DNA-based TLR7/9 antagonist, IMO-3100, has been tested in phase I clinical trials and showed promising results for RA. All these prove that targeting TLRs or their activation represents an attractive therapeutic option for RA.

However, we should notice that there are different types of TLRs in RA as demonstrated in our study. They might interact or work cooperatively with each other during the disease. Targeting one specific TLR might not suffice for RA amelioration. More indepth studies should be taken to further elucidate the pathogenic roles of TLRs in RA and the potential of targeting them for overcoming the chronic and persistent disease.
